# Plant growth-promoting endophytic bacteria versus pathogenic infections: an example of *Bacillus amyloliquefaciens* RWL-1 and *Fusarium oxysporum* f. sp. *lycopersici* in tomato

**DOI:** 10.7717/peerj.3107

**Published:** 2017-03-16

**Authors:** Raheem Shahzad, Abdul Latif Khan, Saqib Bilal, Sajjad Asaf, In-Jung Lee

**Affiliations:** 1 School of Applied Biosciences, Kyungpook National University, Daegu, South Korea; 2 Chair of Oman’s Medicinal Plants & Marine Natural Products, University of Nizwa, Nizwa, Oman

**Keywords:** Growth promotion, Hormonal modulation, Amino acid regulation, Endophytic *Bacillus amyloliquefaciens*, *Fusarium oxysporum* infection

## Abstract

Fungal pathogenic attacks are one of the major threats to the growth and productivity of crop plants. Currently, instead of synthetic fungicides, the use of plant growth-promoting bacterial endophytes has been considered intriguingly eco-friendly in nature. Here, we aimed to investigate the in vitro and in vivo antagonistic approach by using seed-borne endophytic *Bacillus amyloliquefaciens* RWL-1 against pathogenic *Fusarium oxysporum* f. sp. *lycopersici*. The results revealed significant suppression of pathogenic fungal growth by *Bacillus amyloliquefaciens* in vitro. Further to this, we inoculated tomato plants with RWL-1 and *F. oxysporum* f. sp. *lycopersici* in the root zone. The results showed that the growth attributes and biomass were significantly enhanced by endophytic-inoculation during disease incidence as compared to *F. oxysporum* f. sp. *lycopersici* infected plants. Under pathogenic infection, the RWL-1-applied plants showed increased amino acid metabolism of cell wall related (e.g., aspartic acid, glutamic acid, serine (Ser), and proline (Pro)) as compared to diseased plants. In case of endogenous phytohormones, significantly lower amount of jasmonic acid (JA) and higher amount of salicylic acid (SA) contents was recorded in RWL-1-treated diseased plants. The phytohormones regulation in disease incidences might be correlated with the ability of RWL-1 to produce organic acids (e.g., succinic acid, acetic acid, propionic acid, and citric acid) during the inoculation and infection of tomato plants. The current findings suggest that RWL-1 inoculation promoted and rescued plant growth by modulating defense hormones and regulating amino acids. This suggests that bacterial endophytes could be used for possible control of *F. oxysporum* f. sp. *lycopersici* in an eco-friendly way.

## Introduction

Plant growth and productivity is strongly affected by the associated microbiota in the soil. These microbial resources can influence the fitness and survival of plants, either beneficially or antagonistically (Bardgett & van der Putten, 2014). Soil-borne plant pathogens are hazardous to the plant growth and productivity (Gajbhiye et al., 2010). Most soil-borne pathogens survive in soil for long periods of time where they remain dormant until they find a suitable host (Vurro & Gressel, 2006). Some key pathogenic fungi are the species from the genus *Fusarium*. Particularly, *Fusarium oxysporum* is a pervasive soil-borne phytopathogen that can cause serious diseases such as vascular wilt, root rot, and damping off in many plants (McGovern, 2015). Tomato is one of the most important crops sensitive to such infections worldwide, and is especially sensitive to vascular wilt by *F. oxysporum* (Inami et al., 2014). *F. oxysporum* percolate inside the root epidermis, colonizes the roots, occupies the stele, and finally attacks xylem vessels which cause yellowing, shriveling, and finally the death of an infected plant (Olivain & Alabouvette, 1999).

Along with all the alternatives available in the agronomic industries, fungicides play a valuable role in controlling plant diseases; however, their application can cause serious environmental problems and encourage resistance in some fungi (Zouari et al., 2016). Combating the antagonistic behavior of pathogenic fungi can also be achieved through microbial enemy control strategies. Biological control through plant growth-promoting rhizobacteria or endophytic bacteria offers an eco-friendly alternative to chemically synthesized fungicides for pathogenic fungal attacks (Droby et al., 2009). Microorganism with plant growth-promoting potential reprograms the growth of their associated host, thus influencing physiology and phytohormonal signaling during pathogenic attacks (Kloepper, Ryu & Zhang, 2004; Rosenblueth & Martínez-Romero, 2006). They are also known to help host plants by combating the adverse implications of wide range of physiochemical stresses just as salinity, osmotic, and heavy metal (Choudhary et al., 2016; Kang, Radhakrishnan & Lee, 2015; Saleem et al., 2007). Simultaneously, in plant growth improving microbes, endophytes have recently been coined for their intriguingly interesting role in mitigating biotic stresses. There are a few examples recently reported for counteracting pathogenic disease incidence as shown by Waqas et al. (2015), Eljounaidi, Lee & Bae (2016), and Sarangi & Ramakrishnan (2016).

Endophytes refer to the endosymbionts living inside plant tissues without damaging and causing any disease; they can be isolated from inside plant tissues via strict disinfection methods (Arnold & Lutzoni, 2007; Khan et al., 2015). Endophytes live in a completely safe and protected environment, as compared to organisms living in the rhizosphere and phyllosphere (Andrews, 1992). These can be distributed in the rhizosphere (roots), phylloplane (in leaves), laimosphere caulosphere (stems), carposphere (fruits), spermosphere (seeds), and anthosphere (flowers) (Schulz et al., 2002; Arnold & Lutzoni, 2007). Seed-borne endophytes are important for the vertical transmission of endophytes (Kaga et al., 2009). The consequences of seed endophytes have not been fully scrutinized, but their potential to promote plant growth and ameliorate abiotic and biotic stresses have been confirmed based on phytohormone production and nutrient attainment (Shahzad et al., 2016; Xu et al., 2014; Sundaramoorthy & Balabaskar, 2013).

Plants initiate the essential and secondary metabolism response of various plant pathogen associations and their immediate involvement in response to various pathogenic attacks cannot be denied (Mason et al., 2016). Among various essential metabolites, the regulation of amino acids has a particular role in plant resistance (Waqas et al., 2015). Among secondary metabolites, plant hormones assume a dynamic role in plant development and counter biotic stresses. Salicylic and jasmonic (SA and JA) acids are especially involved in mediating stress reactions in plants (Tsuda & Katagiri, 2010). Looking at the prospects of endophytic microbial application to crop disease resistance, in the current study, we aimed to evaluate the in vitro and in vivo antifungal capability of *Bacillus amyloliquefaciens* RWL-1 against *F. oxysporum* f. sp. *lycopersici*, and furthermore to locate the potential mechanism concerned with the bio-control of *F. oxysporum* f. sp. *lycopersici* concerning phytohormonal modulation and amino acid regulation in tomato plants. Previously, *Bacillus amyloliquefaciens* RWL-1 had been isolated from rice seed and it was reported for phytohormone production and plant growth-promoting potential (Shahzad et al., 2016). Our initial analysis showed that the endophytic bacteria produce physiologically active gibberellic acids (GAs) GA_4_, GA_12_, and GA_20_. In addition, the inoculation of this strain significantly promoted various growth attributes of the rice plants through endogenous hormonal modulation and its actively root-colonizing capability.

## Materials and Methods

### Microbial growth conditions

*Bacillus amyloliquefaciens* RWL-1 was isolated previously isolated from rice seeds and was reported for phytohormone production and growth promotion in our previous study (Shahzad et al., 2016). In this study, RWL-1 was grown in Luria–Bertani (LB) media. The pathogenic *F. oxysporum* f. sp. *lycopersici* strain (KACC 40032) was obtained from the Korean Agricultural Culture Collection (KACC, http://genebank.rda.go.kr) and was regrown on potato dextrose agar (PDA) plates at 28 °C for 7 days.

### Quantification of organic acid

The evaluation of organic acid in a culture medium of RWL-1 was carried out according to the method described by Waqas et al. (2015). Briefly, the cultural filtrate was passed out by using 0.22-μm-syringe filter and 20 μL was subjected into the HPLC column of Water Co. (600 E model, included reflective index detector, RI model 410). In the isocratic condition for HPLC, 0.005 m H_2_SO_4_ mobile phases, 0.6 mL/min flow rate and 63 °C temperature was retained (7.7 × 300 mm PL Hi-Plex H column).

### In vitro antifungal assay

**Figure 1:**
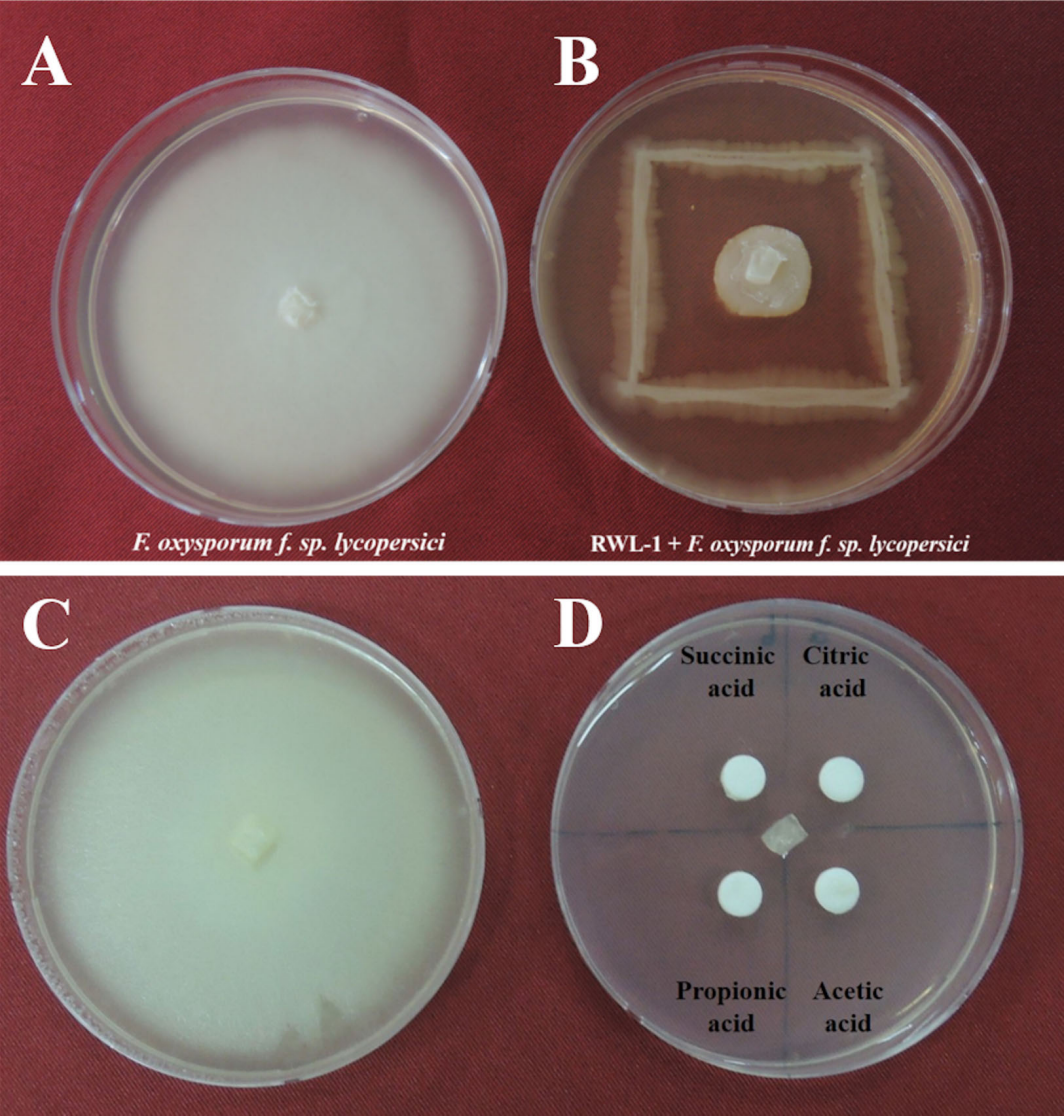
Growth inhibition of endophytic *B. amyloliquefaciens Fusarium oxysporum*. Water control of *Fusarium oxysporum* f. sp. *lycopersici*. (A) Growth inhibition of endophytic *B. amyloliquefaciens* RWL-1 against *Fusarium oxysporum* f. sp. *lycopersici*. (B) Since the *B. amyloliquefaciens* RWL-1 was producing organic acid, we also tested the effect of exogenous organic acids on growth inhibition of *F. oxysporum* f. sp. *lycopersici* (D) in comparison with water control (C). The pictogram is representative of five replications.

The in vitro antagonistic activity of RWL-1 against *F. oxysporum* f. sp. *lycopersici* was measured in a dual culture. Briefly, 0.5 cm^2^ of *F. oxysporum* f. sp. *lycopersici* active mycelia disc was placed at the center of 90 mm petri plate containing freshly prepared LB agar medium. Furthermore, RWL-1 was streaked on the LB agar medium as shown in (Fig. 1B). For untreated plates 0.5 cm^2^ of *F. oxysporum* f. sp. *lycopersici* active mycelial disc was placed at LB agar medium but sterile double distilled water was used instead of bacteria and plates were incubated at 28 °C for 1 week. The experiment was replicated five times and the zone of inhibition was measured according to the following formula described in Kaiser et al. (2005) to examine the antagonistic activity of RWL-1 as compared to a normal *F. oxysporum* f. sp. *lycopersici* growth.

$${\rm{Inhibition }}\;\% = \left( {{\rm{Diameter\;of\;control}} - {\rm{Diameter\;of\;treatment}}} \right) \times {\rm{1}}00$$

### Biological control experiment on tomato plants

Biological control assay was carried out on tomato plants *c.v Yegwang*. For the bio assay, substrate (peat moss (10–15%), coco peat (45–50%), perlite (35–40%), zeolite (6–8%) was used, which contained NO_3_^−^ ∼0.205 mg/g, NH_4_^+^ ∼0.09 mg/g, K_2_O ∼0.1 mg/g, and P_2_O_5_ ∼0.35 mg/g) of TBT (Soil and Fertilizer Technology, Korea) was autoclaved three times to ensure complete disinfection. The tomato seeds were kept in an incubator for 5 days after surface sterilization with 2.5% sodium hypochlorite. Equal size germinated seeds were moved to germination trays for 1 week, and then after 1 week equal size seedlings were shifted to big pots with six plants per treatment in a triplicate experiment. To encourage the plant and endophytic bacterial association, 10 mL RWL-1 (4 × 10^8^ CFU) was applied 5 days before the inoculation of the phytopathogenic fungus. The disease causing the *F. oxysporum* f. sp. *lycopersici* strain (KACC 40032) was grown and maintained on a PDA plate and after seventh day of complete fungal growth, the fungus was cut in equal pieces, applied to scratched root epithelial tissues and covered with soil, while the control was also scratched with no fungal application. The control and treated plants were kept in dark condition at relatively high humidity level of 80% for four day in growth chamber in order to further exploit the pathogenic impact. A total of 14 days after the fungal inoculation, all the growth attributes were recorded and fresh plant biomasses were stored at −70 °C until further analysis.

### Extraction and quantification of amino acid

The extraction and quantification of amino acids were carried out according to the method reported by Khan et al. (2017). Briefly, grounded whole plant samples (100 mg) were hydrolyzed under vacuum in 6N HCl at 110 °C followed by 80 °C for 24 h, respectively. The dried-up remains were homogenized in 0.02N HCl and were passed through a 0.45-μm filter. Furthermore the amino acids were then quantified using automatic amino acid analyzer of Hitachi Japan (L-8900). The experiment was repeated three times and the concentrations were measured by comparison with specific standards.

### Jasmonic acid quantification

Endogenous JA was extracted and quantified according to the protocol described by McCloud & Baldwin (1997). Briefly, the ground freeze-dried whole plant samples (0.3 g) were suspended in extraction solution (70:30 v/v acetone and 50 mm citric acid) and 25 ng JA internal standard ([9, 10-2H^2^]-9, 10-dihydro-JA) was added. To avoid volatile fatty acid losses, the extracts were allowed to evaporate overnight at room temperature. The resulting aqueous solution was filtered and extracted three times with 30 mL diethyl ether. The combined extracts were loaded on a solid-phase extraction cartridge (500 mg of sorbent, aminopropyl). Furthermore, the loaded cartridges were washed with 7.0 mL of trichloromethane and 2-propanol (2:1 v/v). Then, the bound JA and relevant standard were washed with 1 mL of diethyl ether and acetic acid (98:2 v/v). After evaporation, the samples were methylated and were analyzed by GCMS (6890N network GC system), and 5973 network mass selective detector (Agilent Technologies, Palo Alto, CA, USA). To expand the affectability of the method, spectra were recorded in selected ion mode, i.e., in the JA determination case. We inspected the fragment ion at *m/z* = 83 AMU, relating to the base peaks of JA and [9, 10-2H^2^]-9, 10-dihydro-JA. Moreover, the JA was calculated from the value of endo peaks in comparison with their respective standards.

### Salicylic acid quantification

Salicylic acids were extracted and quantified from freeze-dried tomato samples according to the protocol described by Seskar, Shulaev & Raskin (1998). The freeze-dried whole plant tissues (0.2 g) were accordingly extracted with 90% and 100% methanol. The samples were than centrifuged at 10,000×*g* and the combined methanol extract was vacuum-dried. Dried samples were resuspended in 2.5 mL of 5% trichloroacetic acid (TCA) and further partitioned with ethyl acetate, cyclopentane, and isopropanol (ratio of 100:99:1, v/v). The upper organic layer containing free SA was transferred to a 4-mL vial and dried with nitrogen gas. The dry SA was again suspended in 1 mL of 70% methanol and was subjected to HPLC, using a Shimadzu device outfitted with a fluorescence indicator (Shimadzu RF-10AxL) with excitation at 305 nm and emission at 365 nm, filled with a C18 reverse-phase HPLC column (HP Hypersil ODS, particle size 5 μm, pore size 120 Å, Waters). Flow rates of 1.0 mL/min were used.

### Statistical analysis

The triplicate data were from three independent experiments were subjected to Duncan multiple range tests and *t*-tests where appropriate, using 9.2 version SAS software (Cary, NC, USA) and online GraphPad Prism, respectively. The graphs were drawn by using 5.0 version GraphPad Prism (San Diego, CA, USA).

## Results

### In vitro antifungal assay

The potential of *Bacillus amyloliquefaciens* RWL-1 to inhibit the growth of *F. oxysporum* f. sp. *lycopersici* was assessed using dual culture technique (Fig. 1). The results revealed that RWL-1 exhibited a broad spectrum of growth inhibition activity against *F. oxysporum* f. sp. *lycopersici*, resulting in 79.19 ± 3.8 inhibition percentage as compared to control (Fig. 1).

### Organic acid production by RWL-1

**Figure 2:**
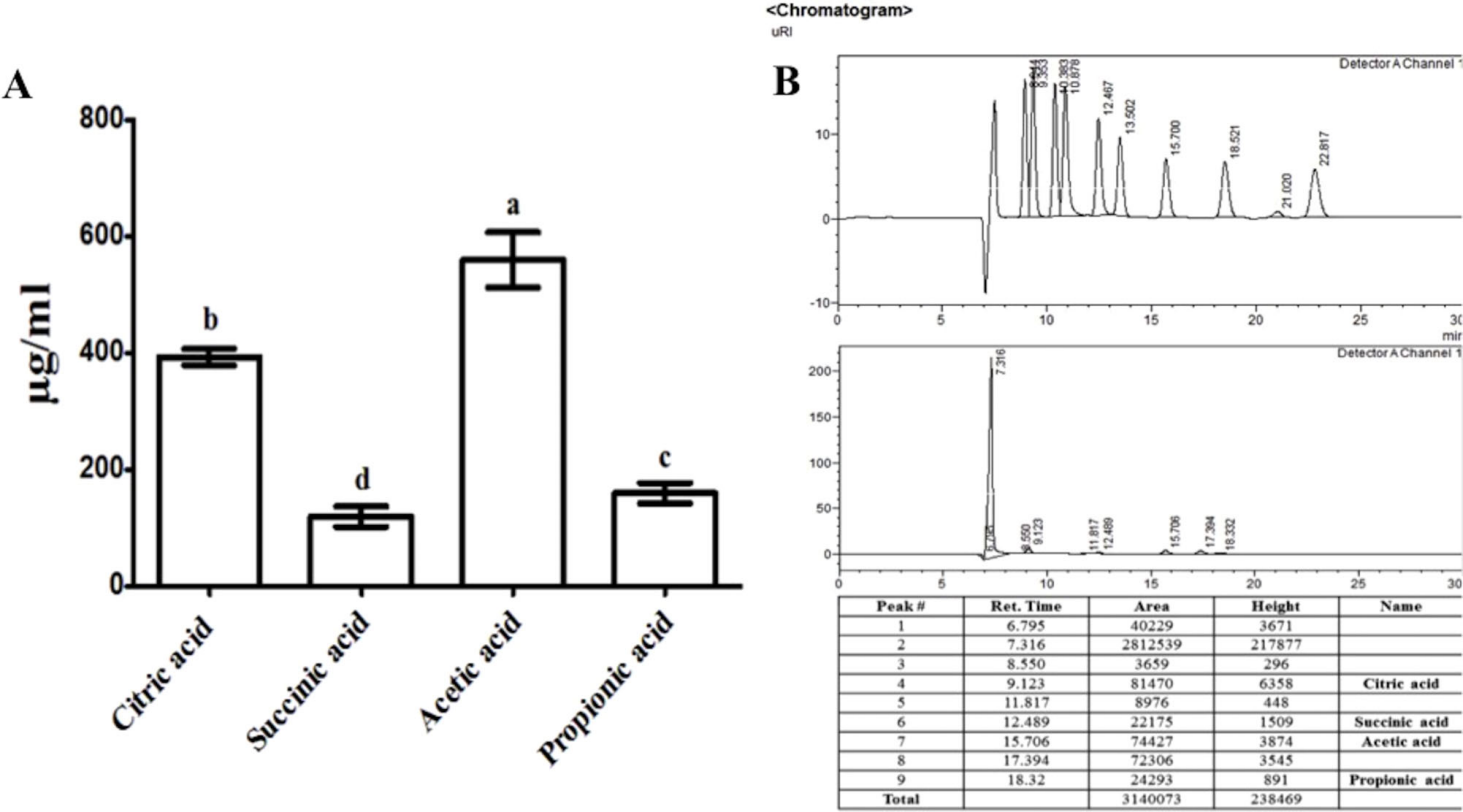
Organic acid secretion by *B. amyloliquefaciens* RWL-1. (A) The four different organic acids were quantified using HPLC and compared with known standards. (B) Each value represents mean ± SD of three replicates. Bars with different letters are significantly different at *P* ≤ 0.05 based on Duncan multiple range test.

The organic acids present in the culture filtrate of RWL-1 were quantified via HPLC. The quantification results revealed that citric acid, succinic acid, propionic acid, and acetic acid were present as well as detectable. The amount of acetic acid was significantly higher (560 ± 81.85 μg/mL) than that of the other acids and was followed by citric acid (393.33 ± 25.17 μg/mL), propionic acid (160 ± 30 μg/mL), and succinic acid (120 ± 30 μg/mL) (Fig. 2). A similar concentration of organic acids as produced by RWL-1 was applied to *F. oxysporum* f. sp. *lycopersici* in LB agar plate, which showed significantly high (*P* < 0.05) suppression of the pathogenic fungus (Fig. 1).

### *Bacillus amyloliquefaciens* RWL-1 ameliorative response to tomato growth during disease incidence

**Figure 3:**
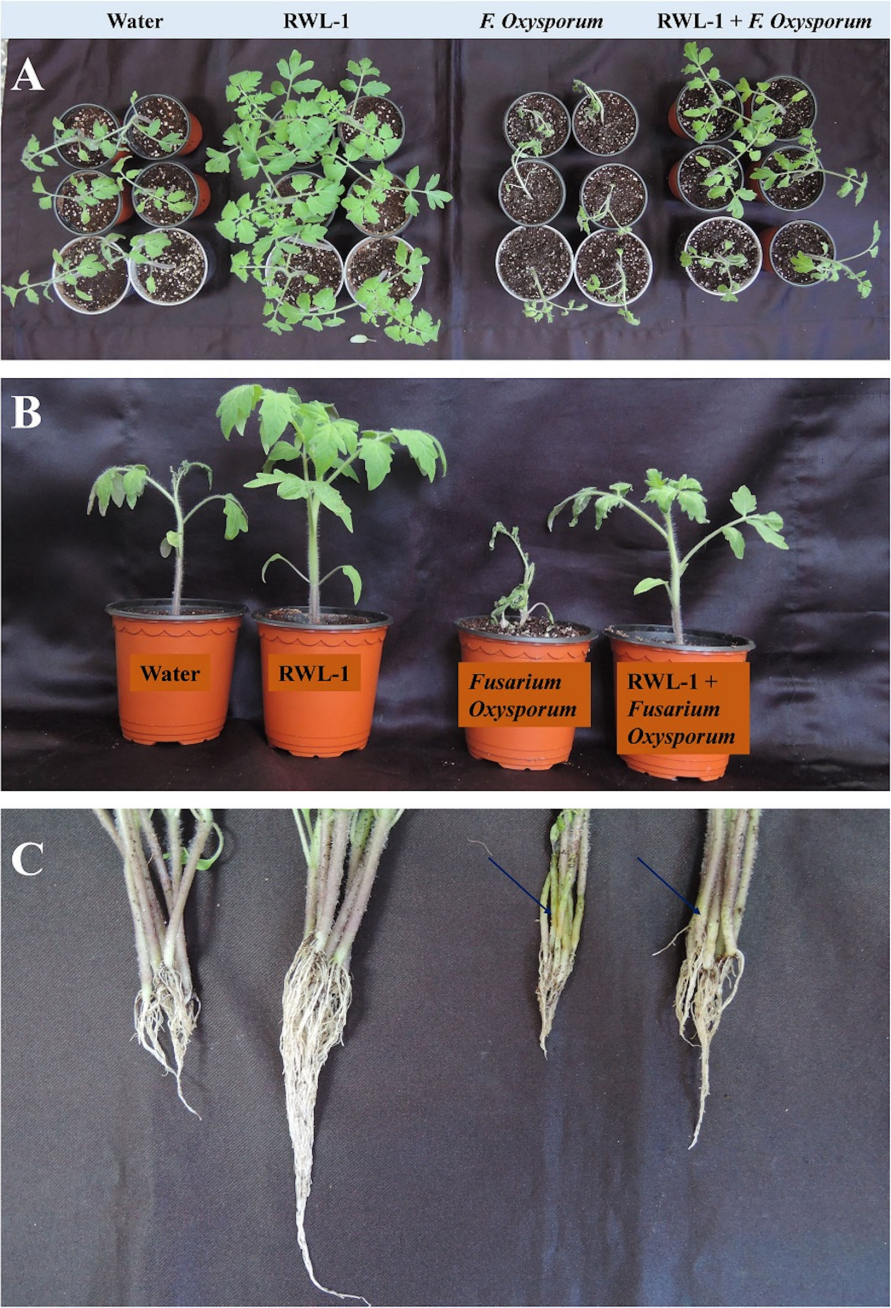
Pathogenic effect of *F. oxysporum* f. sp. *lycopersici* on tomato plant inoculated with RWL-1. (A) The aerial view and effects of RWL-1 inoculation under control condition and *F. oxysporum* f. sp. *lycopersici* infection. (B) The effect of RWL-1 inoculation and *F. oxysporum* f. sp. *lycopersici* infection. (C) The intensity of damage caused by *F. oxysporum* f. sp. *lycopersici* in the roots/stem diameter.

In order to judge the bio-control efficiency of RWL-1, in vivo experiments were carried out against *F. oxysporum* f. sp. *lycopersici* in tomato plants. The plants were treated with water prior to *F. oxysporum* f. sp. *lycopersici* inoculation for disease development. The disease symptoms by *F. oxysporum* f. sp. *lycopersici* were continually increased throughout the experiment and plants died after 2 weeks of disease incidence. The plant roots treated with RWL-1 cells before *F. oxysporum* f. sp. *lycopersici* inoculation, interestingly improved plant development, dramatically decreased the disease symptoms, and enabled plants to survive as compared to sole disease treatments (Fig. 3; Table 1).

**Table 1:** Influence of *B. amyloliquefaciens* RWL-1 on growth promoting traits of tomato under normal and biotic stress conditions.

Treatment	S.L. (cm)	R.L. (cm)	S.F.W. (g)	S.D.W. (g)	C. C. (SPAD)
**Control**	17.71 ± 0.81b	4.07 ± 0.53b	10.74 ± 0.71b	0.53 ± 0.06b	26.79 ± 2.28b
**RWL-1**	22.71 ± 1.52a	10.14 ± 0.63a	30.79 ± 2.76a	1.46 ± 0.04a	34.19 ± 1.53a
***Fusarium oxysporum* f. sp. *lycopersici***	13.83 ± 0.78b	2.21 ± 0.27b	7.31 ± 0.54b	0.38 ± 0.01b	13.47 ± 1.98b
**RWL-1 + *Fusarium oxysporum* f. sp. *lycopersici***	17.57 ± 1.02a	4.21 ± 0.64a	12.78 ± 0.24a	0.73 ± 0.04a	24.76 ± 0.73a

**Notes:**

S.L., Shoot length; R.L., Root length; S.F.W., Seedlings fresh weight; S.D.W., Seedling dry weight; C.C., Chlorophyll content.

Each value represents mean ± SD of 12 replicates from three independent experiments.

Values in columns followed by different letters are significantly different at *P* ≤ 0.05.

This result indicates that RWL-1 inoculation significantly improved all the growth attributes in non-diseased plants as well as in diseased plants. In case of non-diseased plants, the RWL-1 inoculation to plants maximized the shoot length (28.23%), root length (149.14%), fresh and dried weights (168.68% and 175.47%), and chlorophyll contents (27.62%) in comparison with their respective controls (Table 1). A similar tendency of improved growth attributes were noted in diseased plants as well; the RWL-1-treated plants before *F. oxysporum* f. sp. *lycopersici* inoculation showed significantly improved shoot and root length (27.04% and 90.49%), fresh and dry weight (74.82% and 92.10%), and chlorophyll contents (83.81%) in comparison with sole inoculation of *F. oxysporum* f. sp. *lycopersici* (Table 1; Fig. 3).

### Defense-related endogenous phytohormonal regulation

#### Jasmonic acid contents of RWL-1-inoculated diseased plants

**Figure 4:**
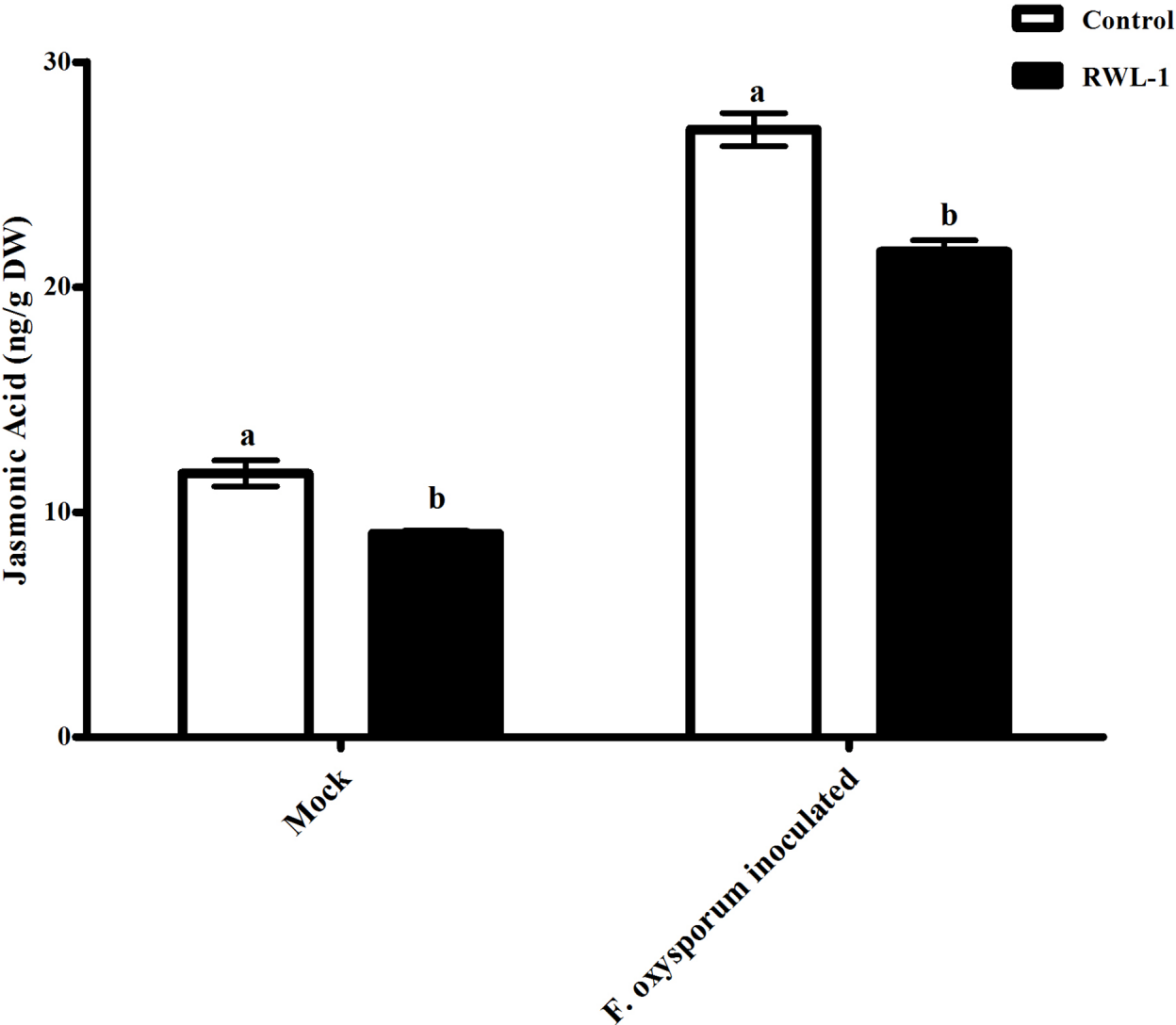
Regulation of endogenous JA under endophytic (*B. amyloliquefaciens* RWL-1) and pathogenic infection (*F. oxysporum* f. sp. *lycopersici*). Each value represents mean ± SD of six replicates from three independent experiments. Bars with different letters are significantly different at *P* ≤ 0.05 based on *t*-test.

The plants treated with endophytic RWL-1 cells showed significantly reduced amount of endogenous JA contents as compared to those in non-endophytic associated plants. A similar trend was seen for pathogenic attacks. During *F. oxysporum* f. sp. *lycopersici* infection, the RWL-1 cell-treated plants before *F. oxysporum* f. sp. *lycopersici* inoculation showed significantly reduced endogenous JA contents as compared to those that had been given a sole inoculation of *F. oxysporum* f. sp. *lycopersici* (Fig. 4). In non-diseased plants, significantly higher amounts (11.72 ± 0.58) of endogenous contents were found in DW-treated plants in comparison to those in RWL-1 treated plants (9.05 ± 0.13). In diseased plants, *F. oxysporum* f. sp. *lycopersici* inoculation significantly increased the amount of endogenous JA (27 ± 0.74), but the RWL-1 treatment before *F. oxysporum* f. sp. *lycopersici* inoculation significantly reduced endogenous JA content (21.58 ± 0.49; Fig. 4).

#### Salicylic acid contents of RWL-1 inoculated diseased plants

**Figure 5:**
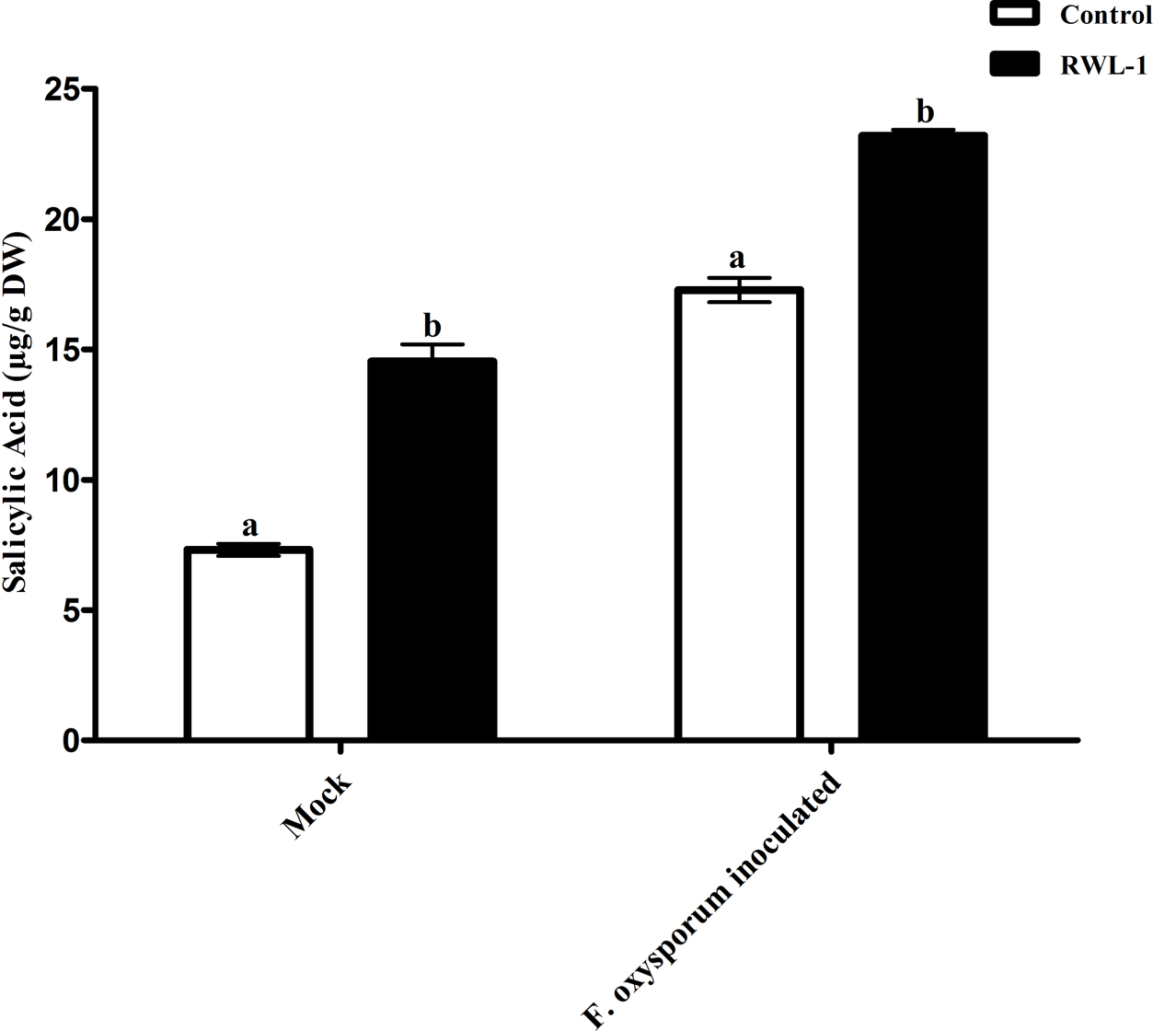
Regulation of endogenous salicylic acid under endophytic (*B. amyloliquefaciens* RWL-1) and pathogenic infection (*F. oxysporum* f. sp. *lycopersici*). Each value represents mean ± SD of six replicates from three independent experiments. Bars with different letters are significantly different at *P* ≤ 0.05 based on *t*-test.

The significantly increased level of endogenous SA contents were recorded in RWL-1 treated plants in comparison to those in DW-treated plants, while, during pathogenic attack, the *F. oxysporum* f. sp. *lycopersici* inoculation significantly lowered the endogenous SA contents as compared to plant roots treated with RWL-1 cells before *F. oxysporum* f. sp. *lycopersici* inoculation (Fig. 5). In non-pathogenic interactions, the RWL-1 cell-treated plants showed significantly higher amounts (14.54 ± 0.65) of endogenous SA in comparison with DW-treated plants (7.31 ± 0.23). As in the pathogenic interactions, higher amounts of endogenous SA content (23.20 ± 0.22) were observed in plants treated with RWL-1 cells before *F. oxysporum* f. sp. *lycopersici* inoculation, while a significantly decreased amount of endogenous SA (17.28 ± 0.47) was found in plants solely inoculated with *F. oxysporum* f. sp. *lycopersici* (Fig. 5).

#### Amino acids regulation in diseased plants inoculated with RWL-1

The amino acids asparagine (Asp), alanine (Ala), leucine (Leu), arginine (Arg), threonine (Thr), methionine (Met), serine (Ser), phenylalanine (Phe), tyrosine (Tyr), cysteine (Cys), valine (Val), isoleucine (Ile), glutamine (Glu), histidine (His), lysine (Lys), and proline (Pro) were measured using an amino acid analyzer for all the treatments (Table 2). Under normal conditions, the RWL-1-inoculated plants revealed higher amino acid contents in comparison with DW-treated plants (Table 2). All of the amino acids were considerably increased in RWL-1-treated plants compared to DW-treated plants, except Cys, which showed no significant difference. The results further confirmed that under control conditions, RWL-1 inoculation increased the aspartic acid (33.83%), Thr (40.98%), Ser (47.77%), glutamic acid (43.19%), glycine (37.48%), Ala (36.99%), Val (35.61%), Met (13%), Ile (34.06%), Leu (35.20%), Phe (37.48%), Lys (42.89%), His (35.92%), Arg (10.54%), and Pro (30.82%) contents in comparison with the respective control (Table 2).

**Table 2:** Regulation of Amino acids (μg/g DW) in the tomato plants inoculated with *B. amyloliquefaciens* RWL-1 under normal conditions and *F. oxysporum* f. sp. *lycopersici* infection.

Treatment	Asp	Thr	Met	ILE	Ser	Glu	Leu	Tyr	Gly	Phe	Lys	Cys	Val	His	Arg	Ala	Pro
**Control**	138.63 ± 1.72b	30.29 ± 3.38b	12.04 ± 1.16b	154.55 ± 1.97b	45.18 ± 1.87b	205.64 ± 5.33b	339.44 ± 3.33b	16.65 ± 1.35a	121.48 ± 0.48b	141.08 ± 2.63b	107.88 ± 1.99b	5.06 ± 0.29a	98.04 ± 2.00b	49.54 ± 1.62b	152.46 ± 2.72b	202.60 ± 3.53b	101.94 ± 2.93b
**RWL-1**	185.52 ± 3.21a	42.70 ± 2.60a	13.63 ± 0.16a	207.18 ± 2.41a	66.76 ± 1.71a	294.44 ± 4.17a	458.89 ± 5.01a	7.26 ± 0.17b	167.00 ± 3.41a	184.73 ± 3.89a	154.14 ± 3.34a	5.45 ± 0.37a	132.95 ± 2.47a	67.33 ± 2.41a	168.52 ± 1.74a	277.53 ± 1.33a	133.35 ± 1.59a
***Fusarium oxysporum* f. sp. *lycopersici***	60.41 ± 2.56b	13.50 ± 2.08b	4.28 ± 0.93b	68.89 ± 4.48b	9.77 ± 0.94b	35.38 ± 3.05b	165.26 ± 3.98b	19.93 ± 1.06b	57.98 ± 1.89b	73.67 ± 2.59b	51.31 ± 2.66b	10.50 ± 1.33a	49.33 ± 1.91b	20.80 ± 3.37b	71.45 ± 1.45b	179.44 ± 0.81b	58.66 ± 4.28b
**RWL-1+ *Fusarium oxysporum* f. sp. *lycopersici***	194.60 ± 4.03a	45.45 ± 2.59a	11.21 ± 1.23a	186.67 ± 3.16a	63.43 ± 1.88a	281.85 ± 3.85a	388.35 ± 4.29a	27.91 ± 0.96a	147.66 ± 2.16a	166.83 ± 2.70a	134.39 ± 3.68a	6.28 ± 0.12b	121.88 ± 2.07a	60.00 ± 2.05a	167.06 ± 2.23a	234.77 ± 3.09a	109.44 ± 1.77a

**Notes:**

Asp, Aspartic acid; Thr, Threonine; Met, Methionine; ILE, Isoleucine; Ser, Serine; Glu, Glutamic acid; Leu, Leucine; Tyr, Tyrosine; Gly, Glycine; Phe, Phenylalanine; Lys, Lysine; Cys, Cysteine; Val, Valine; His, Histidine; Arg, Arginine; Ala, Alanine; Pro, Proline.

Each value represents mean ± SD of three independent experiments.

Values in columns followed by different letters are significantly different at *P* ≤ 0.05.

Under pathogenic attack, RWL-1 treatment before *F. oxysporum* f. sp. *lycopersici* inoculation resulted in significantly higher amounts of aspartic acid, glutamic acid, Thr, His, Ser, glycine, Ala, Arg, Met, Tyr, Phe, Leu, Ile, Lys, Val, and Pro as compared to those after the sole inoculation of *F. oxysporum* f. sp. *lycopersici*, except in the case of Cys, which showed no significant difference (Table 2). With regards to pathogenic interaction, the results showed that RWL-1 inoculation before *F. oxysporum* f. sp. *lycopersici* infection increased aspartic acid (222.14%), Thr (236.67%), Ser (549.24%), glutamic acid (696.64%), glycine (154.68%), Ala (133.82%), Val (147.08%), Met (161.92%), Ile (170.97%), Leu (135%), Tyr (40.05%), Phe (126.46%), Lys (161.92%), His (188.47%), Arg (133.82%), and Pro (86.57%) contents, while Cys (67.2%) content was decreased in comparison with plants infected with *F. oxysporum* f. sp. *lycopersici*.

## Discussion

Recently, *Bacillus* strains as potent biological control agents for many plant diseases have been reported in various studies (Zhi et al., 2016; Zouari et al., 2016). These studies have suggested that *Bacillus* is easy to cultivate, capable of sporulation, and have a long shelf life. Members of *Bacillus* and *Pseudomonas* are most reported for plant growth promoting and stress mediating seed endophytes (Chaves-López et al., 2015; Sundaramoorthy & Balabaskar, 2013; Choudhary & Johri, 2009). The species belonging to *Bacillus* are known as plant growth promoters that can increase crop growth and productivity (Quan et al., 2006; Gajbhiye et al., 2010). The bio-control potential of *Bacillus licheniformis, Bacillus subtilis, Bacillus cereus, Bacillus pumilus*, and *Bacillus amyloliquefaciens* has been documented in numerous reports (Han et al., 2016; Pane & Zaccardelli, 2015). Because of their bio-fertilizer and bio-control properties, they are turning out to be progressively vital as a natural substitute for chemically integrated pesticides (Qiao et al., 2014). Among various *Bacillus* strains, *Bacillus amyloliquefaciens* showed stronger antagonism than any other studied species. However, a few studies have shown *Bacillus amyloliquefaciens* living in the endophytic mode of life. Endophytic *Bacillus amyloliquefaciens* with an extensive antagonistic activity has been documented (Zouari et al., 2016; Chen et al., 2016; Soares et al., 2015).

Most of the convincing approaches of *Bacillus amyloliquefaciens* for bio-control are root colonization (Fan et al., 2011; Wu et al., 2015) and antibiotic production (Nam et al., 2015). Although the demanding mechanism is not yet clear, our results showed that probable antagonism against *F. oxysporum* f. sp. *lycopersici* infected healthy plants by rapid spore and mycelia propagation in several ways, such as insects, irrigation water, and infected dead plants and can cause discoloration and wilting of vascular tissues, root rot, and damping off of seedlings (Zhao et al., 2014; Lecomte et al., 2016). Subsequently, determination of its control method is difficult (Zhao et al., 2014).

**Figure 6:**
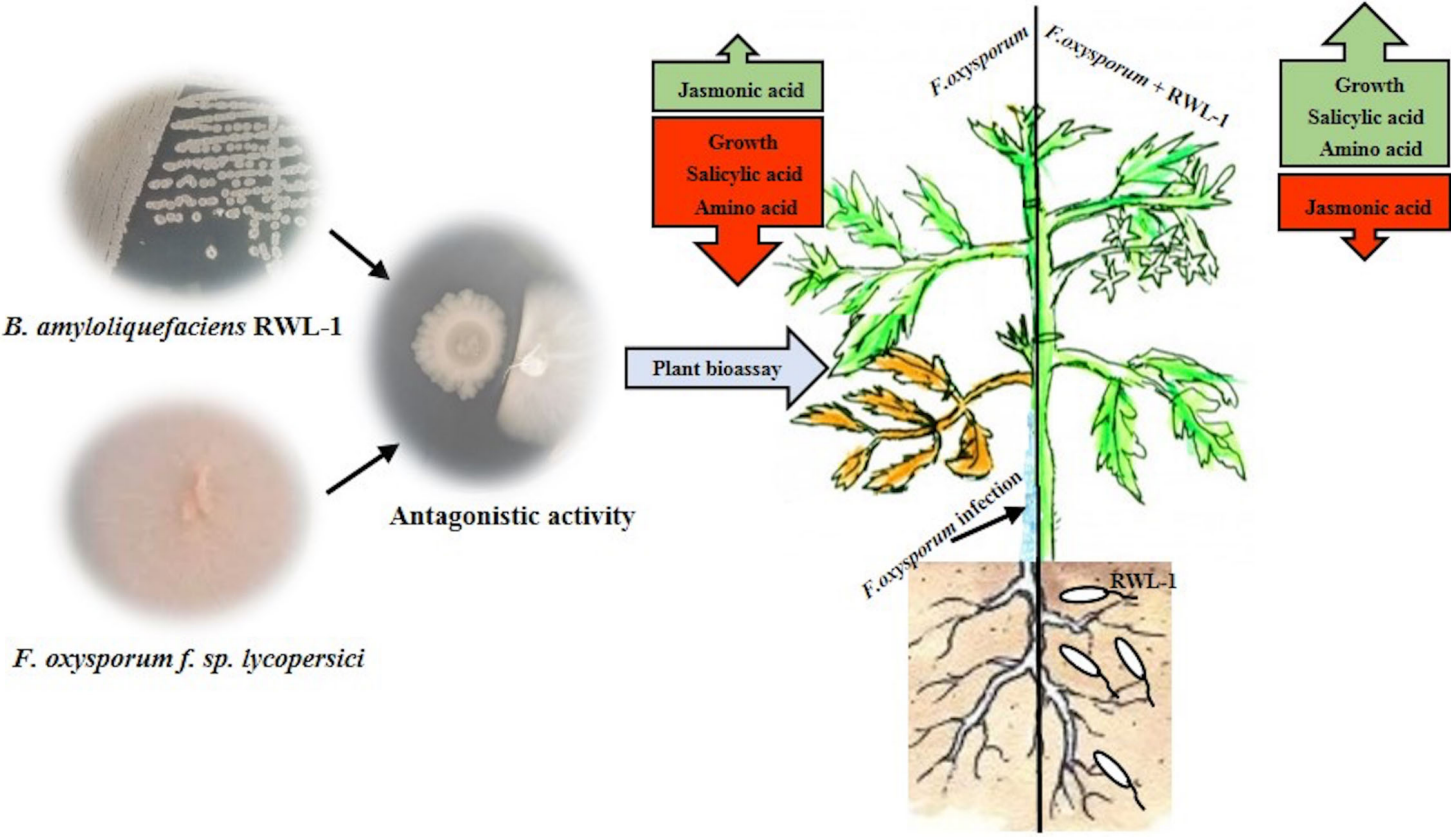
In vitro antagonistic activity of RWL-1 against *F. oxysporum* f. sp. *lycopersici* and understanding the influence of RWL-1 on the survival of tomato plants under pathogenic *F. oxysporum* f. sp. *lycopersici* infection. RWL-1 was applied to plants to measure its effects on morphology with reference to amino acid regulation and defense hormonal modulation under diseased attack.

The growth-promoting capability of RWL-1 was reported previously (Shahzad et al., 2016). In this experiment, the growth-promoting effect of RWL-1 was reconfirmed (Fig. 3; Table 1). *Bacillus amyloliquefaciens* produced a range of secondary metabolites, which is considered important for the improvement of plant growth and amelioration of various biotic and abiotic stresses (Srivastava et al., 2016; Wang et al., 2016; Chen et al., 2007). In this study, it was shown that the gibberellins and organic acid-producing potential of RWL-1 offer extra assistance to plants, and enhancement in plant growth can induce resistance to various biotic and abiotic stresses (Shahzad et al., 2016). In terms of biotic and abiotic stresses, such bacterial endophytes can ameliorate salinity, drought and temperature stress and can improve resistance against pathogenic attack (Fig. 6).

Many researchers have reported disease mitigation with inoculation of various plant growth-promoting bacterial endophytes and more specifically with inoculation of *Bacillus amyloliquefaciens* (Kröber et al., 2016; Chen et al., 2016). Therefore, in this study, it was presumed that the inoculation of *Bacillus amyloliquefaciens* RWL-1 mitigated the deleterious effect of *F. oxysporum* f. sp. *lycopersici* disease to the root zone of tomato plants. Before *F. oxysporum* f. sp. *lycopersici* infection, plant roots were pretreated with cells of RWL-1, and the inoculation not only reduced the disease rigorousness and mitigated the disease symptoms, but also promoted the plant’s growth, which suggests interference with early infection processes that further resulted in limitation of disease development (Mei & Flinn, 2010).

In this study, during pathogenic infection, endophytic association mitigated the disease and improved the growth and biomass of tomato; this may be due to inhibition of pathogenic infection, high nutrient uptake and promotion of plant growth (Ongena & Jacques, 2008). Similarly, the current findings are in accordance with the results of numerous scientists, who have reported similar results of plant promotion, in vitro and in vivo inhibition, and bio-control of various pathogenic fungal diseases such as damping-off of soybean (Yu et al., 2002), root wilt of tomato (Vitullo et al., 2012), anthracnose of strawberry (Yamamoto, Shiraishi & Suzuki, 2015), green mold and blue mold rot of citrus (Hao et al., 2011), Fusarium wilt of banana (Wang et al., 2013), ring rot of apple (Chen et al., 2016), and charcoal rot of soybean and common bean (Torres et al., 2016) by *Bacillus amyloliquefaciens*.

Our results also suggest that microbial strains producing bioactive constituents can help the inoculated plant to reduce the negative impacts of pathogenesis and abiotic stresses. Vassilev, Vassileva & Nikolaeva (2006) elucidated this simultaneous role of bacteria and their biocontrol activity. The author suggested that production of phosphate solubilization inoculums could help the host plant to combat disease incidence of *F. oxysporum*. Similar conclusions were also drawn by Servin (2004), suggesting that bacterial populations producing bioactive constituents can assist plants to counteract disease-induced stress. The results of our study also conform to those of the previous findings that organic acid-like constituents can help relieve plants from the effects of diseases. Waqas et al. (2015) also shown that endophytes-producing siderophores and organic acids are helpful in combating pathogenic effects in sunflower plants. Such ameliorative effects are usually predominated by endogenous hormonal regulators such as JA and SA.

In this study, we found that RWL-1 inoculation extensively modulated endogenous plant defense hormones, i.e., JA and SA, in comparison with control tomato plants, with and without pathogenic infection caused by *F. oxysporum* f. sp. *lycopersici*. Similar results of increased endogenous SA and decreased endogenous JA with the application of plant growth-promoting microbes were reported by Khan et al. (2015), Waqas et al. (2015), and Shahzad et al. (2016), suggesting the role of SA in induced systemic resistance (Zhang et al., 2002; Pozo & Azcón-Aguilar, 2007). Resistance against the phytopathogenic fungus attack was induced on the basis of endogenous JA and SA contents, demonstrating the positive role of endophytes against pathogenic fungi (Schouten, 2016; Spoel, Johnson & Dong, 2007; Halim et al., 2006). These can act as phytoalexins during pathogenic interactions. A recent study by Siciliano et al. (2015) suggested that high JA, SA, and abscisic acid (ABA) could counteract *Fusarium* responses in rice plants. This study also showed that RWL-1 inoculation activated the endogenous physiological apparatus to influence the disease-causing ability of *F. oxysporum* f. sp. *lycopersici*. Although there are numerous studies suggesting that cross-talk exists between JA, ABA, and SA, our understanding is still limited in terms of beneficial endophytic bacterial species such as those producing phytohormones and organic acids. In addition, such pathogenesis responses can influence the basic machinery of the effected plant; i.e., their essential amino acid metabolism (Fig. 6).

Pathogenesis often contributes to altering amino acid metabolism, for example, in the case of glutamates as shown by Seifi et al. (2013). The authors concluded that alterations in host ammonic acid metabolism in response to various pathogenic situations seem to work in two contradicting ways: (i) by sponsorship the progressing protection procedure to at last shape a productive resistance response, or (ii) being exploited by the pathogen to advance and encourage disease. The results of the amino acid analysis performed in this study are in agreement with the first proposal, as the results showed the significant beneficial effect of RWL-1 toward plant growth and disease resistance by activating the amino acid biosynthesis. Various important amino acids viz. aspartic acid, Ser, glutamic acid, and Pro, were significantly enhanced in RWL-1 inoculated plants in comparison with those in non-inoculated plants when pathogenic infection was caused. Kamoun (2006) showed that a high frequency of Ser, Thr, and Ala within the Pep-13 motif are important for activation of plant defenses during pathogenesis. Antão & Malcata (2005) suggested that plant-originated Ser is essential to activate plant defenses against *F. oxysporum* infections. Jones & Jones (1997) have emphasized on the importance of Leu rich motif repeats can improve plant defenses. These previous reports support our result as well, where we found a high concentrations of Ser, Leu, and Pro. Whereas, Pro and/or hydroproline have been credited for strengthening the cell wall during pathogenic attacks (Rashid, 2016). Similar results were recorded by Rojas et al. (2014), where they have extensively discussed the potential benefits of primary metabolism activation during pathogenic stresses. Zeier (2013) also suggested a similar point of view regarding the active role of amino acids in enhancing plant immune responses. Similar results of increased amino acids were previously reported in response to various stresses (Dulermo et al., 2009; Pratelli & Pilot, 2014).

Our results demonstrated the organic acid production and in vitro and in vivo antagonism of RWL-1 against *F. oxysporum* f. sp. *lycopersici*. The RWL-1 not only promoted the tomato growth but also induced resistance against the serious disease-causing pathogenic *F. oxysporum* f. sp. *lycopersici*. The endogenous hormonal modulation and amino acid regulation under normal and pathogenic attack may have activated the resistance against pathogenic fungus. The bacterial endophytes secrete a number of secondary metabolites, which can induce resistance in the plants against various biotic and abiotic stresses (Fig. 6). Therefore, further studies are needed to ascertain secondary metabolites produced by bacterial entophytes and determine their role in plant defense against pathogenic infection-induced stresses.

## Supplemental Information

10.7717/peerj.3107/supp-1 Supplemental Information 1 Raw data for growth dynamics of all treatments.

10.7717/peerj.3107/supp-2 Supplemental Information 2 JA and SA quanitification.

10.7717/peerj.3107/supp-3 Supplemental Information 3 Organic acid quantification.

10.7717/peerj.3107/supp-4 Supplemental Information 4 Endogenous amino acid regulation.

## Additional Information and Declarations

### DNA Deposition

The following information was supplied regarding the deposition of DNA sequences: KR677384; https://www.ncbi.nlm.nih.gov/nuccore/937424470.

### Data Deposition

The following information was supplied regarding data availability: The raw data has been supplied as Supplemental Dataset Files.
